# Vitamin D Status and Factors Associated with Vitamin D Deficiency during the First Year of Life in Preterm Infants

**DOI:** 10.3390/nu13062019

**Published:** 2021-06-11

**Authors:** Jae-Hun Jung, Eun-Ah Kim, Sang-Yoon Lee, Jung-Eun Moon, Eun-Joo Lee, Sook-Hyun Park

**Affiliations:** 1 Department of Pediatrics, School of Medicine, Kyungpook National University, Daegu 41404, Korea; wogns7602@gmail.com (J.-H.J.); dmsk0328@naver.com (E.-A.K.); gguggusy@gmail.com (S.-Y.L.); subuya@hanmail.net (J.-E.M.); pshmom00@gmail.com (E.-J.L.); 2 Pediatrics, Kyungpook National University, Chilgok Hospital, Daegu 41404, Korea

**Keywords:** vitamin D deficiency, preterm infants, Korea

## Abstract

We aimed to investigate the changes in vitamin D levels and factors associated with vitamin D deficiency (VDD) during the first year of life in Korean preterm infants. We enrolled 333 preterm infants who were born at Kyungpook National University Children’s Hospital between March 2013 and December 2019. 25-hydroxyvitamin D (25-OHD) levels and medical records were collected at birth, 6 months, and 12 months of age. The mean gestational age was 33.4 ± 2.3 weeks and mean 25-OHD levels at birth were 18.2 ± 13.5 ng/mL. The incidence of VDD was 82.8%, 30.6%, and 27.0% at birth, 6 months, and 12 months, respectively. The incidence of severe VDD (25-OHD < 10 ng/mL) was 31.5%, 1.5%, and 0%, at birth, 6 months, and 12 months, respectively. Among infants with severe VDD, the deficiency persisted in 49.6% at 6 months, and 35.3% at 12 months. The strongest predictor of VDD during follow-up was 25-OHD concentration at birth. Vitamin D supplementation at 400 IU/day did not affect vitamin D levels during the first year of life. Therefore, it is important to prevent neonatal VDD through maternal vitamin D supplementation during pregnancy. Further research is needed to determine the optimal vitamin D supplementation dose for Korean preterm infants.

## 1. Introduction

Vitamin D is an important micronutrient involved in the skeletal and immune systems and neuromuscular functioning [1]. It also plays a regulatory role in several inflammatory processes [2]. Several clinical and animal studies have reported that vitamin D supplementation may reduce respiratory tract infections and vitamin D levels are associated with neonatal morbidities such as respiratory distress syndrome and bronchopulmonary dysplasia [3,4,5]. Low cord blood levels of vitamin D increase the risk of sepsis and respiratory tract infections during the first year of life [6,7,8]. In addition to these immune aspects, vitamin D is essential for normal calcium metabolism and skeletal growth, both crucial processes for preterm infants [9,10]. Bone formation and mineralization occur mainly in later pregnancy, and are, therefore, affected by both prematurity and inadequate maternal mineral supplementation during pregnancy [11]. Low nutrient levels in preterm infants can increase the risk of metabolic bone disease, such as osteopenia of prematurity. This may be caused by insufficient calcium and phosphorus intake or absorption and low vitamin D levels [11,12].

The strongest factor determining vitamin D levels in the fetus and newborns is maternal vitamin D levels during pregnancy [13,14]. Low maternal vitamin D levels predicted infant vitamin D deficiency (VDD) at birth; however, normal maternal vitamin D levels were also found in infants with VDD at birth [13]. South Korea has one of the lowest vitamin D levels in women [15]. In one Korean study of pregnant women, three-quarters had VDD and one-quarter of those with VDD had severe deficiency (25-hydroxyvitamin D (25-OHD) levels < 10 ng/mL) [16]. Among Korean preterm infants, Park et al. found VDD in 98.9% of the study sample, and half had severe VDD at birth [17].

To our knowledge, no studies have been conducted to record changes in vitamin D levels in preterm infants with severe VDD at birth in Korea. This study aimed to investigate the changes in 25-OHD levels during the first year of life and clarify the factors associated with vitamin D status and deficiency in Korean preterm infants. To our knowledge, this is the first study of this nature in this population.

## 2. Materials and Methods

### 2.1. Study Population

We performed a retrospective medical records review of a total of 871 preterm infants who were born at Kyungpook National University Hospital (latitude 35.87° N), Korea, from March 2013 to December 2019. Overall, 538 infants were excluded due to major congenital anomaly, chromosomal abnormality, and insufficient data on their 25-OHD levels within 24 h after birth. A total of 333 preterm infants were included, and vitamin D levels were available in 268 infants at 6 months of age. The study was approved by the Institutional Review Board of Kyungpook National University Chilgok Hospital, with a waiver of informed consent (KNUCH 2020-10-009).

Patient demographic and clinical data were obtained by manual chart review. Maternal demographic and clinical characteristics included maternal age, delivery mode, and maternal disease, such as gestational diabetes, premature rupture of membranes (PROM), and pregnancy-induced hypertension (PIH) or preeclampsia. Neonatal characteristics at birth were gestational age, birth weight, head circumference, and 25-OHD levels. During follow-up, the collected data included the z-score of the body weight and head circumference, 25-OHD levels, feeding type, and vitamin D supplementation.

All preterm infants received 400 IU of vitamin D per day from birth until discharge. After discharge, information about vitamin D supplementation was provided to caregivers of infants who were fed with breast milk exclusively, or received less than 1 L per day of formula milk, or infants diagnosed with VDD at 6 or 12 months according to the guideline of Korean Society of Neonatology. [18].

### 2.2. Laboratory Analysis

The serum 25-OHD levels were obtained from cord blood or blood samples within 24 h of birth. During follow-up, we used radioimmunoassay (DIAsource 25OH-Vit.D3-Ria-CT kit, DIAsource ImmunoAssays S.A., Louvain-la-Neuve, Belgium) to analyze 25-OHD levels from blood samples obtained at 6 and 12 months. In our study, we categorized vitamin D levels of <30 ng/mL as VDD and 25-OHD levels of ≥30 ng/mL as vitamin D sufficiency. Severe VDD was defined as 25-OHD levels of <10 ng/mL.

### 2.3. Statistical Analysis

We used SPSS Statistics software version 26.0 for statistical analysis (IBM Corp., Armonk, NY, USA). The Shapiro–Wilk test was performed for checking normal distribution. Student’s *t*-test or the chi-square test was used to analyze continuous or categorical variables respectively. Logistic regression analysis was used to adjust for potential confounders associated with 25-OHD levels. A *p*-value < 0.05 was considered statistically significant.

## 3. Results

### 3.1. Demographic and Clinical Characteristics of the Population

**Figure 1:**
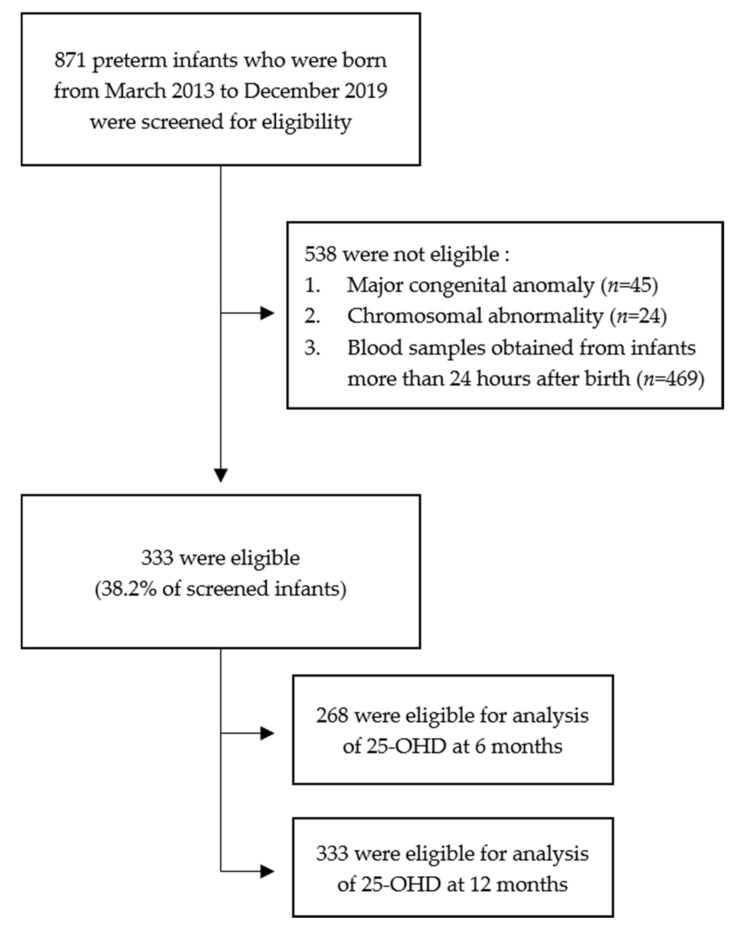
Flow chart of the study populations.

Figure 1 shows the flow chart of the study population. Overall, 871 infants were screened for eligibility. In total, 538 infants were excluded based on the following criteria: major congenital anomaly, chromosomal abnormality, blood sample obtained >24 h after birth, or gestational age of >37 weeks. In this study, a total of 333 preterm infants were eligible.

Table 1 shows the clinical and demographic characteristics of the included 333 preterm infants. The mean gestational age was 33.4 ± 2.3 weeks and mean birth weight was 1989.4 ± 497.5 g. The mean 25-OHD levels at birth were 18.2 ± 13.5 ng/mL, and the incidence rates of VDD and severe VDD at birth were 82.8% and 31.5%, respectively. Among infants with severe VDD, the deficiency persisted in 49.6% at 6 months and 35.3% at 12 months. At 6 months, the mean 25-OHD levels were 39.7 ± 16.2 ng/mL, and 30.6% of the preterm infants had VDD. At 12 months, the mean 25-OHD levels were 38.6 ± 14.1 ng/mL and 27% of the preterm infants were vitamin D deficient.

### 3.2. Comparison of Neonatal Clinical and Demographic Factors between Infants with and Without Vitamin D Deficiency

Table 2 shows the differences in the distribution of clinical characteristics between the VDD group and the nonVDD group. At birth, the VDD infants had lower gestational age (33.2 ± 2.4 weeks) than the nonVDD infants (34.0 ± 1.9 weeks, *p* = 0.02). The VDD group had a significantly higher proportion of female infants than the nonVDD group. Significant differences in birth weight and delivery mode were observed between the VDD and nonVDD infants, but maternal clinical factors and birth season were not statistically different between the two groups.

At six months, the nonVDD infants had a lower mean gestational age (33.2 ± 2.4 weeks) than the VDD infants (33.8 ± 2.0 weeks, *p* = 0.039). There were significant differences in 25-OHD levels at birth between the VDD (13.3 ± 12.1 ng/mL) and nonVDD infants (20.9 ± 19.4 ng/mL, *P* = 0.001). Regarding feeding, the nonVDD group had a higher proportion of infants fed with formula milk than the VDD group. No statistical differences in birth season, body weight, and vitamin D supplementation were observed between the two groups.

At 12 months, the mean serum 25-OHD level was 23.9 ± 4.8 ng/mL in the VDD group and 44.1 ± 12.4 ng/mL (*P* < 0.001) in the nonVDD group. The mean serum level of 25-OHD at birth was significantly lower in the VDD group (14.6 ± 11.5 ng/mL) than the nonVDD group (21.2 ± 19.0 ng/mL, *P* = 0.002). The nonVDD group had more infants fed with formula milk compared with those fed with breast milk at 12 months. Moreover, in the nonVDD group, most of the infants were delivered via cesarean section compared with the VDD group. However, no significant differences in gestational age, birth weight, SGA, body weight at 12 months, season, and vitamin D supplementation were observed between the two groups. During the one-year follow-up, no statistically significant differences in the maternal history of gestational diabetes, hypertension, and PROM, were observed between the two groups.

### 3.3. Risk Factors for VDD at Birth and Follow-Up

As the gestational age increased, the risk of VDD at birth decreased (OR, 0.715; 95% CI, 0.547–0.934; P = 0.014, Table 3). Female infants had a higher incidence of VDD at birth (OR, 2.34; 95% CI, 1.219–4.492; P = 0.011). The incidence of VDD was lower in infants delivered via cesarean section than normal vaginal delivery (OR, 0.262; 95% CI, 0.094–0.733; P = 0.011). Maternal clinical factors, including gestational diabetes, hypertension, and PROM, were not associated with neonatal VDD at birth.

Higher 25-OHD concentration at birth was significantly correlated with a lower incidence of VDD at 6 months (OR, 0.952; 95% CI, 0.926–0.979; *P* < 0.001, Table 4). Infants fed with formula milk had lower risk of VDD than infants fed with breast milk (OR, 0.318; 95% CI, 0.136–0.746; *P* = 0.008). Gestational age, birth weight, seasonal variation, and vitamin D supplementation were not statistically associated with VDD at six months.

At 12 months, a higher 25-OHD concentration at birth was associated with a lower incidence of VDD (OR, 0.969; 95% CI, 0.947–0.992; *P* = 0.009, Table 5). Gestational age, birth weight, season, feeding type, and vitamin D supplementation were not correlated with VDD at 12 months.

## 4. Discussion

In this study, more than four out of five preterm infants were diagnosed with VDD at birth, which is consistent with previous Korean studies [17,19,20]. Although the incidence of VDD decreased during the 1-year follow-up, VDD persisted in over 30% of infants at 6 months and 27% at 12 months. Nearly a third of infants had severe VDD at birth, of whom, over half recovered by 6 months, and nearly two-thirds recovered by 12 months of age. However, this left more than a third of infants with severe VDD at birth with ongoing VDD at 1 year, despite vitamin D supplementation. Higher 25-OHD levels at birth were significantly associated with higher 25-OHD levels at 6 and 12 months. Formula feeding was associated with lower risk of VDD at 6 months but not at 12 months. Season of birth and vitamin D supplementation were not correlated with vitamin D levels during follow-up.

Similar to previous work [21], we found that infants with VDD at birth had a lower gestational age than nonVDD infants. This is explained by the fact that preterm infants have lesser time than full-term infants to accumulate vitamin D from the mother through placental transfer [22]. Recent studies have shown that maternal VDD during pregnancy may be associated with an increased risk of preterm birth and the infant being small for gestational age [23,24,25]. However, in this study, being small for gestational age was not associated with VDD at birth.

VDD rates were significantly higher in female infants at birth, which is consistent with the findings of previous studies [20]. However, this gender association was not found in the adjusted results at 6 and 12 months. Differences such as progression of weaning to solid food and vitamin D synthesis through outdoor activities may have contributed to these results in this study. According to a study of adolescents in Korea, VDD is one of the most common health problems, with rates of 98.9% in boys and 100% in girls [26]. While there have been no longitudinal studies on changes in vitamin D levels from birth to adulthood, it is of concern that VDD persisting through adolescence and into pregnancy may then affect neonatal vitamin D status.

Previous studies have shown a significant increase in VDD risk for infants delivered via cesarean section [27]. In contrast, preterm infants in our study delivered by cesarean section had a lower incidence of VDD and higher 25-OHD levels at birth compared with those born by normal vaginal delivery. One possible explanation for the conflicting results in our study is that our hospital is a tertiary hospital with a large proportion of high-risk mothers and a high rate of cesarean delivery and, therefore, not representative of the general maternity population. These factors may have influenced the outcomes in this study.

Infants who were fed with formula milk had a lower prevalence of VDD at follow-up; however, after adjusting for confounders, the correlation was not evident at 12 months. Preterm infants, especially those who are only breastfed, need supplementation of vitamin D due to a possible inadequate intake of maternal and dietary vitamin D [28,29]. The American Academy of Pediatrics recommends that infants receive at least 400 IU/day of vitamin D within the first few days of life [12,30]. In this study, vitamin D supplementation was not associated with VDD at follow-up. One possible reason for this unexpected finding is that data on the regularity and frequency of vitamin D supplementation were not available from the medical records. In our hospital, 400 IU/day of vitamin D was administered to preterm infants in the first few days of life. There is currently no consensus recommendation for the dose of vitamin D in preterm infants, but the Korean Pediatric Society also recommends 400 IU per day of vitamin D during infancy. In another Korean study, giving 800 IU per day of vitamin D was found to improve vitamin D levels during hospitalization in very low birth weight infants with severe VDD at birth [31]. Therefore, a 400-IU/day dose of vitamin D may have been insufficient to address VDD in preterm infants. At the 12 months follow-up, no statistically significant correlation was observed between the type of feeding and VDD. The main source of vitamin D is cholesterol, which is converted into vitamin D in the skin and requires ultraviolet light exposure [30]. Most parents are advised to keep their newborns out of direct sunlight for the first few months of life; therefore, vitamin D levels during this period are largely dependent on dietary vitamin D [32,33]. In this study, the duration of sun exposure was not included because of a lack of information. In addition to increased sun exposure, infants start to eat weaning food at 4–6 months of corrected age and then gradually start solid food. Weaning food not only introduces new types of food and ways of eating but also satisfies nutritional needs, including iron and vitamins. Vitamin D levels in infants are increased by sun exposure and vitamin D from weaning food. These factors may act as confounding factors in this study.

This study has several limitations. First, almost all participants were from Daegu City, Korea. Therefore, the data cannot represent Korea as a whole. Another limitation was that no data on maternal 25-OHD levels at delivery were available in the medical records. However, numerous studies have already demonstrated the relationship between maternal vitamin D levels and vitamin D in infants [13,25,34,35]. We were also unable to collect data on sun-exposure time and weaning food. Finally, in this study, only 400-IU/day of vitamin D was administered to preterm infants, which may have been insufficient for their needs.

To our knowledge, this is the first study to follow-up on VDD in preterm infants in Korea. Overall, infant 25-OHD concentration at birth affected the incidence of VDD during the first year of life. Therefore, it is important to maintain sufficient vitamin D levels in pregnant women to promote appropriate neonatal vitamin D levels at birth. Korea has one of the highest prevalences of VDD in preterm infants and pregnant women. The recommended dose of vitamin D failed to improve the vitamin D levels in preterm infants. Therefore, randomized controlled studies are needed to evaluate the appropriate dose of vitamin D in Korean preterm infants.

## 5. Conclusions

During the one-year follow-up in this study, the incidence of VDD and severe VDD in preterm infants decreased. However, one-quarter of preterm infants remained vitamin D deficient, and a third of infants with severe VDD at birth still had VDD at one year of life. The most important predictor of vitamin D levels during the one-year follow-up was the 25-OHD level at birth. Therefore, it is important to prevent VDD through adequate vitamin D supplementation during pregnancy to improve maternal vitamin D levels. Because the recommended 400-IU per day of vitamin D supplementation in preterm infants did not make a significant difference to vitamin D status during follow-up, further research is needed to determine the optimal dosing of vitamin D in Korean preterm infants.

## Figures and Tables

**Table 1:** Characteristics of the study participants.

Infant Characteristics	
Females	159 (47.7)
GA, weeks	33.4 ± 2.3
Body weight, g	1989.4 ± 497.5
Head circumference, cm	30.3 ± 2.5
Cesarean section	260 (78.1)
SGA	28 (8.4)
25-OHD, ng/mL	18.2 ± 13.5
VDD	274 (82.8)
Severe VDD	105 (31.5)
Maternal characteristics	
Maternal age, years	32.4 ± 4.2
Diabetes	39 (11.7)
PIH or preeclampsia	48 (14.4)
PROM	107 (32.1)
At 6 months (*n* = 268)	
Body weight, z-score	1.2 ± 1.1
Head circumference, z-score	1.0 ± 1.8
25-OHD, ng/mL	39.7 ± 16.2
VDD	82 (30.6)
Severe VDD	4 (1.5)
Feeding type	
Breast milk	46 (16.3)
Formula milk	209 (73.9)
Breast milk + formula milk	28 (9.9)
At 12 months (*n* = 333)	
Body weight, z-score	0.2 ± 1.1
Head circumference, z-score	0.0 ± 1.4
25-OHD, ng/mL	38.6 ± 14.1
VDD	90 (27)
Severe VDD	0 (0)
Feeding type	
Breast milk	28 (8.4)
Formula milk	288 (86.7)
Breast milk + formula milk	16 (4.8)

Values are presented as mean ± standard deviation or number (%). Abbreviations: GA, gestational age; SGA, small for gestational age; VDD, vitamin D deficiency; PIH, pregnancy induced hypertension; PROM, premature rupture of membranes; 25-OHD, 25-hydroxyvitamin D.

**Table 2:** Comparison of neonatal clinical and demographic factors between infants with and without vitamin D deficiency.

Factors	At Birth			6 Months			12 Months		
	VDD (*n* = 274)	NonVDD (*n* = 57)	*p* Value	VDD (*n* = 82)	NonVDD (*n* = 186)	*p* Value	VDD (*n* = 90)	NonVDD (*n* = 243)	*p* Value
Female	142 (51.8)	17 (29.8)	0.003	39 (47.6)	97 (52.2)	0.510	42 (46.7)	117 (48.1)	0.902
GA, weeks	33.2 ± 2.4	34.0 ± 1.9	0.020	33.8 ± 2.0	33.2 ± 2.4	0.039	33.6 ± 2.3	33.3 ± 2.3	0.373
Body weight, *z*-score	0.4 ± 1.1	0.1 ± 1.0	0.034	1.1 ± 1.1	1.3 ± 1.2	0.182	0.4 ± 1.1	0.2 ±1.1	0.148
HC, *z*-score	−0.1 ± 1.3	0.0 ± 1.3	0.664	0.9 ± 2.0	1.0 ±1.8	0.489	−0.1 ± 1.4	0.1 ±1.3	0.467
Cesarean section	206 (75.2)	52 (91.2)	0.008	59 (72.0)	146 (78.5)	0.275	58 (64.4)	202 (83.1)	<0.001
SGA	24 (8.8)	4 (7.0)	0.798	6 (7.3)	15 (8.1)	1.000	9 (10.0)	19 (7.8)	0.657
Season			0.065			0.847			0.911
Spring	74 (27.0)	13 (22.8)		25 (30.5)	49 (26.3)		25 (28.1)	61 (25.1)	
Summer	71 (25.9)	21 (36.8)		16 (19.5)	36 (19.4)		20 (22.5)	62 (25.5)	
Fall	69 (25.2)	18 (31.6)		24 (29.3)	54 (29.0)		23 (25.8)	60 (24.7)	
Winter	60 (21.9)	5 (8.8)		17 (20.7)	47 (25.3)		21 (23.6)	60 (24.7)	
Feeding type						0.003			0.002
BM				21 (25.6)	24 (12.9)		12 (13.3)	16 (6.6)	
FM				49 (59.8)	148 (79.6)		69 (76.7)	219 (90.5)	
BM + FM				12 (14.6)	14 (7.5)		9 (10.0)	7 (2.9)	
Vit D Supplement				32 (39.0)	67 (36.0)	0.681	12 (13.1)	34 (14.0)	1.000
Laboratory data									
Calcium	9.5 ± 0.8	9.7 ± 0.8	0.352				10.3 ± 0.6	10.3 ± 0.5	0.787
Phosphorus	5.4 ± 0.9	5.4 ± 0.8	0.904				5.6 ± 0.5	5.8 ± 0.5	0.098
ALP	198.6 ± 66.1	202.8 ± 58.5	0.657				280.8 ± 82.9	294.0 ± 77.1	0.183
25-OHD, ng/mL	13.7 ± 7.3	47.4 ± 24.2	<0.001	22.5 ± 6.1	47.4 ± 13.2	<0.001	23.9 ± 4.8	44.1 ± 12.4	<0.001
25-OHD at birth, ng/mL				13.3 ± 12.1	20.9 ± 19.4	0.001	14.6 ± 11.5	21.2 ± 19.0	0.002

Values are presented as mean ± standard deviation or number (%). Abbreviations: GA, gestational age; HC, head circumference; SGA, small for gestational age; ALP, alkaline phosphatase; 25-OHD, 25-hydroxyvitamin D; BM, breast milk; FM, formula milk; VDD, vitamin D deficiency.

**Table 3:** Risk factors for vitamin D deficiency at birth in preterm infants.

Factors	Adjusted		
	Odds Ratio	95% CI	*P* Value
Gestational age	0.715	0.547–0.934	0.014
Birth weight	1.001	1.000–1.002	0.203
Sex (female)	2.340	1.219–4.492	0.011
Cesarean section	0.262	0.094–0.733	0.011
Small for gestational age	2.622	0.679–10.116	0.162
Gestational diabetes	2.052	0.709–5.933	0.185
Gestational hypertension	1.366	0.515–3.623	0.530
PROM	0.883	0.446–1.749	0.721
Seasonal variation compared to spring			
Summer	0.568	0.252–1.280	0.172
Fall	0.706	0.303–1.646	0.421
Winter	1.698	0.551–5.230	0.357

Abbreviations: Cl, confidence interval; PROM, premature rupture of membranes.

**Table 4:** Risk factors for vitamin D deficiency at six months after birth in preterm infants.

Factors	Adjusted		
	Odds Ratio	95% CI	*p* Value
Gestational age	1.071	0.841–1.364	0.578
Birth weight	1.001	1.000–1.002	0.247
Sex (female)	0.788	0.437–1.422	0.430
Cesarean section	1.259	0.605–2.621	0.538
Small for gestational age	1.054	0.299–3.714	0.934
Gestational diabetes	0.926	0.366–2.345	0.871
Gestational hypertension	0.938	0.374–2.349	0.891
PROM	1.358	0.723–2.551	0.342
Vitamin D status at birth	0.952	0.926–0.979	<0.001
Vitamin D supplementation	0.889	0.449–1.760	0.736
Season variation compared to spring			
Summer	0.619	0.260–1.473	0.378
Fall	0.576	0.268–1.237	0.157
Winter	0.489	0.213–1.121	0.091
Compared to breast milk			
Formula milk	0.318	0.136–0.746	0.008
Breast milk + formula milk	0.827	0.284–2.410	0.728

Abbreviations: Cl, confidence interval; PROM, premature rupture of membranes.

**Table 5:** Risk factors for vitamin D deficiency at 12 months after birth in preterm infants.

Factors	Adjusted		
	Odds Ratio	95% CI	*p* Value
Gestational Age	1.139	0.917–1.413	0.239
Birth weight	1.000	0.999–1.001	0.432
Sex (female)	0.805	0.466–1.392	0.438
Cesarean section	0.559	0.297–1.051	0.071
Small for gestational age	1.407	0.483–4.103	0.531
Gestational diabetes	0.687	0.283–1.665	0.406
Gestational hypertension	0.765	0.328–1.787	0.536
PROM	1.261	0.711–2.239	0.428
Vitamin D at birth	0.969	0.947–0.992	0.009
Vitamin D supplementation	0.738	0.311–1.754	0.492
Seasonal variation compared to spring			
Summer	0.778	0.372–1.625	0.503
Fall	1.056	0.505–2.209	0.884
Winter	1.050	0.498–2.212	0.898
Compared to breast milk			
Formula milk	0.505	0.191–1.334	0.168
Breast milk + formula milk	1.730	0.437–6.846	0.435

Abbreviations: Cl, confidence interval; PROM, premature rupture of membranes.
